# A Collaborative Approach to Collecting Data on Children’s Oral Health and Weight Status, Georgia, 2005

**Published:** 2008-03-15

**Authors:** Dafna Kanny, Matthew D Falb

**Affiliations:** Georgia Department of Human Resources, Division of Public Health; Georgia Department of Human Resources, Division of Public Health, Atlanta, Georgia

Throughout life, maintaining good oral health and a healthful weight are important factors in ensuring a good quality of life and limiting risk for disease. However, in Georgia, surveillance systems have done little to monitor the oral health and weight status of children. To fill this gap in surveillance data, Georgia's dental health staff and the nutrition and physical activity staff collaborated to screen third-grade children for height, weight, and dental health. To do so, we added height and weight measurements to the Association of State and Territorial Dental Directors' (ASTDD's) basic screening protocol and used the adapted protocol for the 2005 Georgia Third Grade Survey. The Georgia Department of Human Resources, Division of Public Health, collaborated with the ASTDD to modify the screening protocol. To use their funds as efficiently as possible, two state programs joined forces to collect data, although each program had a different outcome of interest. This joint data collection effort was a significant step in planning public health programs to address poor oral health and obesity among children in Georgia efficiently and inexpensively. Our approach is one that could also work well in other states.

## Introduction

Good oral health and a healthful weight are essential components of general good health throughout life. However, in the United States, dental decay is one of the most common chronic infectious diseases, and the rate of childhood obesity is on the rise. Poor oral health is associated with altered facial appearance, problems with speech and eating, negative self-esteem, poor social interaction, low level of education, low career achievement, and poor emotional health (1). Obesity increases risk for type 2 diabetes, hypertension, sleep apnea, and low self-esteem (2).

The oral health and weight status of adults in Georgia are monitored through the Georgia Behavioral Risk Factor Surveillance System. However, limited data are collected on the oral health and weight status of the state's elementary schoolchildren. The most recent data on the oral health status of the state's elementary schoolchildren were collected 19 years ago through the 1989 Georgia Dental Disease Prevalence Survey (3). Data on the weight status of Georgia's elementary schoolchildren were last collected 6 years ago through the 2002 Georgia Childhood Overweight Prevalence Survey (4).

The purpose of our study was to collect data on the oral health and weight status of Georgia's children. The study's findings will be used to plan the state's oral health promotion program and its nutrition and physical activity initiative.

## Methods

### Data collection and collaboration

In 2004, the state's program to promote oral health was awarded a combined grant from the State Oral Health Collaborative Systems and the federal Title V Special Projects of Regional and National Significance. The objectives of the grant were 1) to develop and support activities that would strengthen infrastructure within the state and community dental networks and 2) to build new state and community systems to care for the oral health of Georgians, especially participants in the Medicaid and PeachCare programs. PeachCare is Georgia's State Children's Health Insurance Program (SCHIP). As a first step to meeting these objectives, the dental professionals in Georgia's Division of Public Health elected to conduct a statewide oral health assessment of third-grade schoolchildren.

Because surveillance data on the weight status of elementary schoolchildren in Georgia were also needed, Georgia's Nutrition and Physical Activity staff elected to partner with the Georgia Oral Health Prevention staff to measure the height and weight of Georgia's elementary schoolchildren at the same time as they were being screened for issues related to oral health. The partnership was innovative in that it modified one established data collection system to meet the data needs for two programs with different outcomes of interest. Collaborating in this way was more efficient than having each program collect data separately. Each program contributed monetary and other resources to purchase data collection equipment, train local public health staff on data collection protocol, and disseminate health promotion materials to schools and families. The Association of State and Territorial Dental Directors (ASTDD) and the Epidemiology Branch, Division of Public Health, Georgia Department of Human Resources provided technical assistance on sampling and analytic methods. The Family Health Branch, Division of Public Health, Georgia Department of Human Resources (the organizational unit that houses both health promotion programs) coordinated the roles and responsibilities of each program.

The disadvantage of the collaboration was a diminished ability to collect detailed data on contributing factors to poor oral health and obesity. Each program would have liked to collect more information on behaviors that increase risk for these conditions, such as poor dental hygiene practices and consumption of sugar-sweetened foods and beverages. We decided, however, to exclude behavioral outcomes in order to increase the likelihood of a sufficient response rate on the parent questionnaire.

Participating children were examined for the presence of caries, untreated dental decay, and dental sealants. If children were in urgent need of dental care, their parents or caregivers were notified and given a referral to a dental professional. Dentists or dental hygienists in each of Georgia's public health districts conducted the oral health screenings. The diagnostic criteria were based on the Association of State and Territorial Dental Directors' (ASTDD's) *Basic Screening Surveys: An Approach to Monitoring Community Oral Health* (5). Before examining the children, all examiners participated in a didactic review of the diagnostic criteria and the oral health screening protocol.

The children's height was measured with Seca 214 portable stadiometers (Hanover, Maryland) and their weight was measured with Tanita HD 351 scales (Arlington Heights, Illinois). The participating children removed their shoes, coats, jackets, sweaters, vests, bags, and hair accessories before being measured. Dentists and dental hygienists from local public health departments weighed and measured the height of participants in 15 of the 17 public health districts included in the screening. Nutritionists from local public health departments measured the height and weight of participants in the two remaining public health districts. The measurements were taken in private areas. Before measuring any children, all examiners were trained in the protocol for measuring height and weight and for calibrating the instruments.

Each child's height and weight measurements were used to calculate body mass index (BMI), which was determined on the basis of sex- and age-specific BMI categories in growth charts developed by the Centers for Disease Control and Prevention (CDC) (5).

Additional data were collected on participating children through a questionnaire administered to their parents or caregivers (6). Items requested on the questionnaire included the child's demographic characteristics, data on dental insurance, use of the dental care system, barriers to dental care, and reasons for previous dental visits. The survey was conducted as a routine public health program evaluation and therefore did not require approval from the Georgia Department of Human Resources Institutional Review Board.

### Sampling and participation

The sample frame was constructed using 2002–2003 school enrollment data from the Georgia Department of Education. All public schools with 25 or more students in third grade were eligible. The final sample comprises 1145 public schools. The sample frame was ordered from the lowest to the highest proportion of students eligible for the Free and Reduced Lunch (FRL) program. Epi Info™ (Version 3.2.2, CDC) was used to select randomly a number from 1 through 20: 15 was the number selected. Therefore the 15th school on the list was the first school selected to participate in our study and every 20th school thereafter. If a school declined to participate, a replacement school within the same FRL sampling stratum was randomly selected. Technical support for sampling was provided by ASTDD. Fifty-seven schools were selected for the study and asked to participate: five declined, so five replacement schools were selected.

Parent questionnaires, consent forms, and health education materials about oral health, nutrition, and physical activity were sent home with all students in the participating schools. As an incentive to participate, schools were given oral health education materials for display on bulletin boards and in libraries. The children who participated in the screening received stickers, toothbrushes, and brushing timers.

In March and April 2005, the dental screening and the height and weight measuring were completed. In the selected schools, 6085 students were eligible to participate in the study; 2961 completed the dental screening and height and weight measurement with parental consent (a 49% student participation rate). When the study was complete, data from the parent questionnaire, oral health screening, and height and weight measurements were available on 2326 (38%) of the eligible children.

Statewide prevalence estimates and 95% confidence intervals were calculated with SAS, Version 9.2 (SAS Institute, Cary, North Carolina). Technical support for data analysis was provided by the Epidemiology Branch at the Georgia Division of Public Health.

## Results

Individual and aggregate results from the screenings were reported (7,8). In addition, a letter describing the oral health, height, and weight of participants was sent home with the children for their parents or caregivers. If the children needed urgent dental care, their parents or caregivers were so advised; they were also referred to dental providers. All parents and caregivers were encouraged to discuss their children's height and weight with their primary care physician.

Results from the screening indicate that 56% of the participants had experienced dental caries, 27% had untreated dental decay, 40% had dental sealants, and 24% were obese (7,8). The oral health and weight status of children in Georgia did not meet the *Healthy People 2010* (9) objectives for caries experience (42%), untreated dental decay (21%), dental sealants (50%), or obesity (5%).

## Issues Related to Collaboration on Data Collection

Although pooling resources between internal and external stakeholders to collect data on multiple health outcomes can be an efficient use of resources, several programmatic and analytic issues need to be considered. One issue is the scope of data needed to guide programmatic activities: researchers must find the right balance between the capacity of survey instruments to collect appropriate data, the amount of the data to be collected, and the burden that collecting data has on survey participants. In Georgia, we collected data on the burden of poor oral health and obesity among the state's third-grade schoolchildren. However, little is known about the behavioral determinants of these significant public health problems, and we did not collect any data that would provide us with information on these problems. The efficacy of including behavioral outcomes will be reevaluated during future screenings.

One analytic consideration was selecting the best sampling and analytic methods to meet the needs of programs and stakeholders. ASTDD has developed sampling and analytic protocols and makes them available to states. ASTDD also gives states flexibility in their approach to analyzing and reporting the data. The Georgia survey used the sampling method recommended by ASTDD but opted to customize the analysis and reporting of the dental and body composition data.

Another analytic consideration is how to design questions on race and ethnicity. The parent questionnaire had two questions that asked parents to provide the race and ethnicity of their children. One optional answer was *unknown*. This option created a problem because 13% of parents selected *unknown* for the ethnicity question and an additional 12% refused to answer the question. These responses limited our ability to make inferences about the growing number of Hispanic children and caused us to weight and analyze the data by race-only categories. Alternative ways of asking parents to indicate the race and ethnicity of their children will be explored in future screenings.

## Conclusion

Poor oral health and obesity are significant public health problems among third-grade students in Georgia. Internal collaboration between the state's Oral Health Prevention Program, the Nutrition and Physical Activity Initiative, and the Epidemiology Branch (coupled with external technical support from ASTDD) produced new data on the oral health and weight status of children in Georgia, a population that would benefit from early intervention. These data were collected through a simple, practical, efficient, and inexpensive method. This approach can be replicated by other states. In Georgia, results from the screenings will serve as a guide for combating poor oral health and obesity among elementary schoolchildren.

## Acknowledgments

The Georgia Department of Human Resources, Division of Public Health, received funds from the U.S. Department of Health and Human Services, Health Resources and Services Administration (Grant Number 6H47MC01931-02-01) and the Centers for Disease Control and Prevention (Cooperative Agreement U58/CCU422885) to implement the screening protocol. We thank Thomas E. Duval DDS, MPH, Georgia's State Dental Director; Linda L. Koskela RDH, MPH, Director, Georgia Oral Health Prevention Program; Mara Galic, MHSc, RD, LD, former Project Coordinator, Georgia Nutrition and Physical Activity Initiative; and Kathy Phipps, DrPH, Data Coordinator, Association of State and Territorial Dental Directors, for their work on the study. We especially thank the staff in the local public health dental programs and nutrition services, the principals and teachers at the participating schools, the parents or caregivers of the participating children, and of course, the participating children themselves without whom the study would have been impossible.
